# Is stenting required before retrograde intrarenal surgery with access sheath

**DOI:** 10.4103/0970-1591.56185

**Published:** 2009

**Authors:** P. M. Mahajan, A. S. Padhye, A. A. Bhave, Y. B. Sovani, Y. B. Kshirsagar, S. S. Bapat

**Affiliations:** Dr. Bodhe, Department of Urology, Ratna Memorial Hospital, Maharashtra Medical Research Society, Pune, Maharashtra, India

**Keywords:** Preoperative stenting, retrograde intrarenal surgery

## Abstract

**Background:**

Flexible ureterorenoscopies continue to assume an increasing role in the armamentarium of the endourologist. In many centers around the world, prior stenting is carried out before retrograde intrarenal surgery (RIRS) to passively dilate the ureter, which facilitates passage of a flexible ureteroscope with or without an access sheath. In our series, the first stage of passive dilatation with prior stenting was totally avoided without compromising the success of the procedure.

**Materials and Methods:**

From January 2004 to December 2007, 54 patients with 55 renal units underwent RIRS. The patients were between 28 and 65 years old. All patients had renal stones ranging in size from 8 mm to 22 mm. The mean serum creatinine level was 1.1 mg%. The lower ureter was dilated under ‘C - arm’ fluoroscopy guidance up to 14 FR. An access sheath of 10/12 Fr was passed over the working guide wire. RIRS (7.5/9.3 Fr) was introduced into the access sheath. The stones were fragmented using a holmium laser. The mean operating time was 85 mins (45-130 mins).

**Results:**

In 52 out of 55 renal units (94.5%), a flexible ureteroscope could be passed successfully into the kidney through an access sheath. In 3 of the cases (5.4%), the lower ureter could not be dilated. In these patients, the procedure was staged after passing a 6/26 JJ stent. An X-ray KUB was done at the 3-month follow-up visit. A total of 50 renal units (94.3%) were stone free at the 3-month follow-up visit.

**Conclusion:**

In a majority of the cases, RIRS could be accomplished successfully during the first sitting. Single stage RIRS did not alter the subsequent stone clearance or increase the incidence of morbidity or complications.

## INTRODUCTION

Retrograde intrarenal surgery (RIRS) is gaining popularity as the primary modality for the management of selected renal calculi. In many centers, RIRS is performed in two stages. In the first stage, a double J stent is inserted to passively dilate the ureter. In the second stage, RIRS and stone fragmentation is carried out. We present our data of 55 renal units wherein the first stage of stent insertion was avoided thereby reducing the number of hospital visits and cost. In our series, 52 renal units (94.5%) avoided the first stage of passive dilatation without compromising the success of the procedure.

## MATERIALS AND METHODS

From January 2004 to December 2007, 54 patients with 55 renal units underwent RIRS for stone disease. All patients were investigated as per the institutional protocol. The mean serum creatinine level was 1.1 mg % (0.5–3.7 mg %). One patient had CRF with bilateral upper pole renal calculi with recurrent urinary tract infection. In all patients, an initial cystoscopy with a retrograde uretero - pyelogram was carried out to delineate the anatomy, followed by the insertion of two guide wires into the kidney. The lower ureter was dilated under ‘C- arm’ fluoroscopy guidance up to 14 Fr by ureteral dilator. An access sheath of 10/12 Fr was passed over one of the guide wires. After removal of the working guide wire and stillate, RIRS (STORZ, ACMI DUR- E, ACMI DUR-D) was introduced into the access sheath. The renal anatomy was studied. Stones were visualized and fragmented using a LISA SPHINX Holmium laser (80 watt). A 200-micron laser fiber was used at the power setting between 10 to 20 watts (energy: 1 joule; frequency: 10 to 20 HZ). Stones were fragmented by painting technique to fragment them into fine gravels, thus obviating stone retrieval. At the end of the procedure, the renal pelvis was irrigated to wash out stone dust and the RIRS was removed along with access sheath. Finally, a 6/26 double J (DJ) stent was inserted over the safety guide wire and left indwelling for 3 to 4 weeks. In 3 cases (5.4%), the lower ureter could not be dilated and a DJ stent was inserted. RIRS was carried out after 4 to 15 days. For 1 female patient who had 2 stones in the pelvis and calyx, the pelvic stones were successfully fragmented by rigid ureteroscope followed by RIRS by flexible scope for the caliceal stones. In 2 patients, after successful fragmentation of the pelvic calculi, stones in the lower calyx could not be fragmented as they were lodged in the inaccessible calyx. For all patients, the bladder was drained until the next day and they were discharged after voiding urine satisfactorily. One patient who developed septicemia was discharged on the fourth day after the surgery after controlling the infection. All patients received antibiotic cover as per culture and sensitivity for 10 days. Those patients who had successful stone fragmentation were followed up at 1 month and at 3 months after removal stent with repeat X- ray KUB and ultrasonography. The mean operating time was 85 mins (45–130 mins).

## RESULTS

The male to female ratio was 24:30. The patients' age ranged from 28–65 years old. All had renal stones ranging in size from 8 mm to 22 mm. A total of 24 patients had solitary stones while 30 had multiple stones in the upper, mid, lower calyx, and pelvis [Table 1].

**Table 1:** Stone size and distribution (total renal units 55)

Stone size (mm)	8-22 mm
Stone location	
Upper calyx	16
Middle calyx	22
Lower calyx	17
Pelvic	15
Stone number	
Solitary	24
2 Stones	21
3 or more stones	8
Bilateral stones	1

STENTING: In 52 out of 55 renal units (94.5%), a flexible ureteroscope could be passed into the kidney through an access sheath successfully in a single stage. In 3 cases (5.4%), the lower ureter could not be dilated. In these patients, the procedure was staged after passing a 6/26 JJ stent. The second stage was successfully performed after 4 to 15 days. Single stage RIRS did not alter the subsequent stone clearance.

STONE CLEARANCE [Table 2]: In 2 of the patients, stones could not be accessed as they were lodged in an inaccessible lower calyx. Among these 2 patients, 1 patient had narrow infundibulum and the other had very acute infundibulo-pelvic angle. In the remaining 50 renal units (94.33%), complete stone clearance was achieved at 3 months follow-up [Table 3]. A total of 3 patients had residual stones of 8 mm, which were treated by SWL, and subsequently the patients were stone free. None of the patients developed ureteric stricture at the follow-up of 3 months as judged by ultrasonography. One patient who developed septicemia after the procedure recovered after treatment. No other complications were observed in the rest of the patients.

**Table 2:** Postoperative outcomes

Stone free rate	No. of patients
Upper calyx	16/16 (100%)
Middle calyx	22/22 (100%)
Lower calyx	15/17 (82.3%)
Renal pelvis	15/15 (100%)
Complications	1/55 (<1.81%)

**Table 3:** Follow-up

Follow-up period	3 months	6 months	1 year
Stone free rate	50/53	39/42	30/35
Number of patients lost to follow-up	0	11	18

## DISCUSSION

There has been a shift toward minimally invasive surgery in all surgical subspecialties in recent decades. RIRS represents an area in which there have been numerous advances that have resulted in excellent patient outcomes with low morbidity. Technologic advances such as miniaturization of ureteroscopes, improved video imaging, and the use of the Holmium laser have expanded the indications for ureteroscopy. The entire upper urinary tract can now be accessed for the diagnosis and treatment of many common urologic conditions. Review of published data shows that in many centers prior DJ stenting was carried out to passively dilate the ureter. We present our data of 55 cases of RIRS in 54 patients.

Huffman and associates first described the removal of large ureteral and renal pelvic stones with a rigid rodlens uretroscope and an ultrasonic lithotripter.[1] As advancements in fiber optic technology have facilitated the development of practical flexible ureteroscopes, the therapeutic potential of flexible ureteroscopes has also improved by several design changes, including a larger working channel to allow for the passage of flexible instruments and active tip deflection combined with passive deflection. Digital ‘Chip at the tip’ technology has immensely improved the visual quality of RIRS.

Fuchs and Fuchs reported the first large series of renal calculi treated by ureteroscopy using a flexible deflectable ureteroscope after a 1 to 2-week period of ureteral stent placement.[2] The introduction of flexible actively deflectable ureteroscopes (7.5 French) has permitted more procedures to be performed without routine prior dilatation. In many centers around the world, prior stenting is carried out before RIRS to passively dilate the ureter to facilitate the passage of the access sheath and flexible ureteroscope.[3,4]

Stents are not without potential morbidity. In addition to irritative urinary symptoms and stent related pain, ureteral stents have been associated with sexual disfunction, bacteriuria, and fever.[5,6] Despite these problems, in many centers ureteral stents are used particularly for passive dilatation before planned ureteroscopic procedures.[3,4,7] This has been useful in the pediatric population. Hubert and Palmer evaluated 26 children with ureteral and/or pelvic calculi. After 2 to 8 weeks of prior stenting, a subsequent ureteroscopy was accomplished successfully in all cases. They reported that passive dilation of the ureteral orifice in preparation for ureteroscopy is a straightforward, successful, and beneficial technique in children, with no associated complications.[7] Ronald, *et al*. retrospectively randomized 90 patients to no pre-operative stenting and pre-stenting groups. In their study, they concluded that pre-stenting is associated with a significantly higher stone-free rate and few complications.[3] In our technique of painting the stone by laser energy which produces stone fragments of smaller than 2 mm in size, we did not encounter the need to manually remove the fragments by tipless basket. Though pre-stenting is very useful in children, it is not essential in the adult population. In our study, a flexible ureteroscopy could be accomplished in 52 renal units (94.5%) without prior passive dilatation. The procedure was performed with a satisfactory stone-free rate and minimal complications. There was no injury to the ureter as a follow-up ultrasound revealed no stasis denoting stricture formation. IVU was not carried out in the follow-up as information gained through a properly performed ultrasonography was satisfactory. With advancement in technology and miniaturization of flexible ureteroscopes, active dilatation of the lower ureter is not required.[8,9]

Use of an access sheath is known to decrease the operating time, cost, and complications hence, active dilatation of the lower ureter was carried out in all cases to facilitate a smooth passage of access sheath.[10,11] Manufacturers of the flexible scopes advise the use of the access sheath to prolong the life of scope. Though the principle of passive dilatation still applies in some cases, we found that its routine use is not necessary in each and every case. By omitting the first stage of passive dilatation, considerable savings in the total cost was achieved. A limitation of this study warrants mention, as there is no comparative arm in the present study.

## CONCLUSION

In the majority of cases, RIRS could be accomplished successfully during the first sitting. Single-stage RIRS did not alter the subsequent stone clearance or increase the incidence of morbidity or complications.
